# Perception of susceptibility to the negative outcomes associated with unprotected sex among University of Venda students

**DOI:** 10.12669/pjms.296.4111

**Published:** 2013

**Authors:** Felix Chima Anyanwu, Daniel Ter Goon, Augustine Tugli, Abiodun Olukoga, Lateef O Amusa, M. Lizzy Netshikweta, Babajide Ajao

**Affiliations:** 1 Felix Chima Anyanwu, Department of Public Health, University of Venda, Thohoyandou, South Africa.; 2 Daniel Ter Goon, Centre for Biokinetics, Recreation and Sport Science, University of Venda, Thohoyandou, South Africa.; 3 Augustine Tugli, Department of Public Health, University of Venda, Thohoyandou, South Africa.; 4 Abiodun Olukoga, Department of Public Health, University of Venda, Thohoyandou, South Africa.; 5 Lateef O Amusa, Centre for Biokinetics, Recreation and Sport Science, University of Venda, Thohoyandou, South Africa.; 6 **M.** Lizzy** Netshikweta, **Department of Advanced Nursing, University of Venda, Thohoyandou, South Africa.; 7 Babajide** Ajao, **Department of Public Health, University of Venda, Thohoyandou, South Africa.

**Keywords:** Perception, Susceptibility, Unprotected sex, Negative outcomes

## Abstract

***Objectives***: The purpose of this study was to determine the perception of University of Venda students on their susceptibility to the negative outcomes associated with unprotected sex.

***Methods***: This cross sectional study involved 408 (206 females and 202 males) University of Venda students residing within the university campus. Simple and systematic sampling methods were used to select participants. A 4-likert scaled self-administered questionnaire was used for data collection and a cut-off point of 75% of the total scores was used as criteria for assessing perception.

***Results*** **:** Majority (90.90%) of the participants understood the magnitude and problems posed by HIV and other sexually transmitted infection in the society, 94.60% believe that anyone can be infected with HIV or fall pregnant when exposed to unprotected sex. Majority (87.30%) believed that alcohol consumption while engaging in sexual activities increases the chances of being infected with HIV, other STI or falling pregnant, 92.40% believed that having multiple sexual partnerships increases the risk of being infected with HIV and other sexually transmitted infections. Eighty six percent of participants agreed or strongly agreed that pregnancy could occur with one sexual intercourse in a month and only 68.45% of the participants believed that practising oral sex could expose them to HIV infection (with no significant difference between male and female).

***Conclusion***: Majority of the students showed high perception regarding their susceptibility to the negative outcomes associated with unprotected sex, especially when they consume alcohol while engaging in sexual activities and when having multiple sexual partnerships. Contrastingly, the students demonstrated low perception regarding susceptibility to HIV transmission through the practice of oral sex. There is a need to create awareness on the dangers posed by the practise of oral sex.

## INTRODUCTION

Unprotected sex refers to any act of sexual contact where a partner or partners do not use condoms to protect themselves from pregnancy and sexually transmitted infections (STI).^1^ This risk behaviour has health implications especially among young people who are at high risk for the negative health outcomes associated with unprotected sex, including infection with Human Immunodeficiency Virus (HIV), the associated Acquired Immunodeficiency Syndrome (AIDS) and unwanted pregnancy.^2^ Individuals between the ages of 14–19 years have the highest unwanted pregnancy rates, and the rates of HIV/AIDS among this group is rising.^2^^,^^3^ It is estimated that about 2.5 million of the 200 million women who become pregnant each year are HIV positive.^2^^,^^4^

Young women constitute the fastest growing category of new HIV cases with the greatest increases being among young women who acquired the infection through heterosexual contact.^5^ Almost 50% of HIV infections occur among people younger than 25 years and 25% of new HIV infections occur among people younger than 22 years^5^ and the risk of HIV infection is heightened by the use of hormonal contraception^6^ and the practice of unprotected sex after alcohol consumption.^7^

Engaging in unprotected sexual intercourse, having multiple sexual partners, lacking the skills to correctly and consistently use condoms, including inadequate knowledge about condom use and the unavailability of condoms and perceiving invulnerability have been highlighted as contributing factors to the high rate of HIV infection in this age group.^8^ Despite a high knowledge of sexual risks, fear of HIV and awareness of the protective value of condoms, young men continue to exhibit high risk behaviour.^9^

In Africa, STIs are one of the commonest reasons for adult attendance at outpatient clinics with 69 million new cases per year in a population of 269 million adults between 15–49 years, and this rate is the highest in the world.^10^ Africa is home to two out of every three persons infected with HIV in the world and nearly 20% of South African adults are living with HIV/AIDS.^11^ Although the chance of becoming infected with HIV is almost equal between men and women, it has been shown that women tend to become infected at an early age.^12^ In some parts of Africa, it has been shown that young women aged 15-19 years can be at higher risk of infection as much as two to eight times more than the boys their age ^12^^,^^13^ and women in sexual relationships with men five or more years older are more likely to be HIV infected.^14^

In Kwazulu- Natal (KZN), people are well informed about the causes and ways to prevent HIV and AIDS and they also perceive it as having serious consequences on the health of people and the society.^15^ Yet, HIV prevalence among pregnant women attending government antenatal clinics in KZN increased from 26.5% in 2002 to 29.1% in 2006.^16^

Although programmes that reduce sexual risk behaviour have been identified as playing an important role in improving behavioural outcomes, adolescents continue to be at risk for HIV infection, other STIs, and pregnancy.^5^ It is therefore important to assess the perception of susceptibility of university students regarding the negative outcomes associated with unprotected sex. This present study was designed to determine the perception of University of Venda students on their susceptibility to the negative outcomes associated with unprotected sex.

## METHODS

The study was conducted among students residing within the University of Venda, South Africa. Details regarding the sampling procedure, instrumentation and data collection procedures are reported elsewhere.^17^ Permission to conduct the research was given by the University of Venda, health, safety and research ethics committee: SHS/12/PH/06/0912.

Four hundred and fifteen questionnaires were collected out of 600 (excess questionnaires were distributed in anticipation of a low return rate) distributed, giving a return rate of 69.17%. Seven questionnaires were not properly completed and were excluded from the study, leaving 408 questionnaires for analysis.

Data was analyzed with Statistical Package for Social Sciences (SPSS). Chi-square test was used to compare differences between variables and statistical difference was set at p<0.05.

## RESULTS

A total of 408 students participated in the study with an age range of 15 to 45 years and a mean age of 22.20 (SD= 3.53). Table-I shows that 371 (90.90%) participants agreed or strongly agreed that HIV infection and other sexually transmitted infections are common problems in our society and 386 (94.60%) either agree or strongly agree that anyone can be infected with HIV or fall pregnant when exposed to unprotected sex. On whether alcohol consumption while engaging in sexual activities increases the chance of being infected with HIV, other STI or falling pregnancy, 356 (87.30%) participants either agreed or strongly agreed while 52 (12.70%) disagreed or strongly disagreed. Three hundred and forty seven (85.10%) participants disagreed or strongly disagreed that antibiotics when taken after unprotected sex could prevent the transmission of HIV while only 14.9% believed it was possible.

However, majority (92.40%) of the participants either agreed or strongly agreed that having multiple sexual partners increases the risk of being infected with HIV and other sexually transmitted infections. About two third of the participants believe that practising oral sex could expose one to HIV infection. On whether one could make a baby even with just one sexual intercourse in a month, 86% either agreed or strongly agreed while 14% either disagreed or strongly disagreed.

Almost all (98.06%) of those participants who did not use condoms at their last sexual intercourse agreed or strongly agreed that anyone could be infected with HIV or even fall pregnant when they expose themselves to unprotected sex, and 92.54% of them also agreed or strongly agreed that if they do not use contraception regularly with their partner, chances are high that they could make a baby even when they do not plan to (Table-II).

**Table-I:** Perceived susceptibility to the negative outcomes associated with unprotected sex

*Questions on STI and unwanted pregnancy*	*n (%) N=408*			
	*SA*	*A*	*D*	*SD*
It is common problems in our society.	248(60.80)	123(30.10)	26(6.40)	11(2.70)
Anyone can be affected	319(78.20)	67(16.40)	13(3.20)	9(2.20)
Only meant for a certain people.	23(5.60)	30(7.40)	76(18.60)	279(68.40)
Sex while drunk can increase ones risk	255(62.50)	101(24.80)	31(7.60)	21(5.10)
Antibiotics can prevent HIV	25(6.10)	36(8.80)	97(23.80)	250(61.30)
Multiple sexual partners increases risk	299(73.30)	78(19.10)	13(3.20)	18(4.40)
Oral sex cannot lead to HIV infection.	45(11.00)	86(21.10)	142(34.80)	135(33.10)
Only homosexuals are at risk for STIs.	17(4.20)	50(12.30)	79(19.40)	262(64.20)
One sex cannot cause pregnancy	74(7.10)	28(6.90)	74(18.10)	277(67.90)
Irregularly use of contraception may lead to pregnancy	215(52.70)	144(35.30)	25(6.10)	24(5.90)

SA = Strongly Agree; A = Agree; D = Disagree; SD = Strongly Disagree

**Table-II:** Condom use at last sex and perceived susceptibility

***Statements***		***NA***		***No***		***Yes***		***Total***		
		***N***	***%***	***N***	***%***	***N***	***%***	***N***	***%***	
Anyone can be infected with HIV or even fall pregnant when they expose themselves to unprotected sex.	A	13	18.06	21	20.39	33	14.16	67	16.42	FET =6.458 p= 0.374
	D	2	2.78	1	0.97	10	4.29	13	3.19	
	SA	54	75.00	80	77.67	185	79.40	319	78.19	
	SD	3	4.17	1	0.97	5	2.15	9	2.21	
If I do not use contraception regularly, I could make a baby even when we do not plan to	Condom use at last sex									FET=10.489 p=0.106
		NA		No		Yes		Total		
		N	%	N	%	N	%	N	%	
	A	22	30.56	31	30.10	91	39.06	144	35.29	
	D	8	11.11	3	2.91	14	6.01	25	6.13	
	SA	39	54.17	64	62.14	112	48.07	215	52.70	
	SD	3	4.17	5	4.85	16	6.87	24	5.88	

NA = Not applicable; SA = Strongly Agree; A = Agree; D = Disagree; SD = Strongly Disagree

## DISCUSSION

The knowledge of the magnitude and severity of the problems posed by HIV and other sexually transmitted infections have been studied among urban and rural communities,^18^ and similar to this, the present study found that majority (90.90%) of the participants understood this problem and are aware of its complications on their health. Individual perception of the risks of being affected by the negative outcome of unprotected sex was also high as 94.60% of participants believe that anyone can be infected with HIV or fall pregnant when exposed to unprotected sex. According to a study in Kenya, 28% of women and 27% of men perceived themselves to be at no risk at all for HIV infection, citing that they had only one sexual partner.^19^ Another study in South Africa also found that only a few young women perceived themselves to be at risk of HIV infection as 32.9% of all women in the sample stated that they were at no risk of infection.^20^

The perception regarding the negative effect of alcohol consumption on condom use was high among participants with no significant difference between male and female, majority (87.30%) believed that alcohol consumption while engaging in sexual activities increases the chance of being infected with HIV, other STI or falling pregnant. This is supported by a study conducted among students at the University of Botwana^21^ which suggests that there is an increased vulnerability to HIV infection because of excessive alcohol consumption, resulting to improper and inconsistent use of condoms. It was further reported by the students that when drunk, they are less careful and may engage in sexual risk behaviour such as having unprotected sex.^22^

Multiple sexual partnerships are perceived as high risk behaviour associated with transmission of HIV and other diseases. These partnerships, link people together sexually and the higher the number of people in the society who have more than one partner, the higher the risk of infection.^23^ The present study and a study conducted in Tanzania ^24^ concur to this fact as they found a high percentage of the participants (92.40% and 88.00%, respectively) believing that having multiple sexual partners increases the risk of being infected with HIV and other sexually transmitted infections.

It is possible for HIV transmission to occur through performing or receiving oral sex^25^ although this is not a common mode of transmission. However, the findings from the present study found that only about two thirds of the participants believed that practicing oral sex could expose them to HIV infection (with no significant difference between male and female). This figure is higher than that described among university students in Nigeria^26^ where less than half (49%) believed that HIV can be transmitted through oral sex.

The perception that one could get pregnant with one sexual intercourse in a month was high among participants (86%). This contrasts with other studies that found many participants believing that they could not get pregnant by having sex just once; arguing that one sex in a month was not enough to make them conceive.^27^

Young people continue to engage in risky sexual behaviour even when they are aware of the consequences and the risk posed to their own lives.^26^ The present study found that among the sexually active participants, 86.67% (26 individuals) of those who consumed alcohol before or during their last sexual intercourse also believed that being drunk while engaging in sexual activities increases ones risk of being infected with HIV and other STIs or even fall pregnant.

## CONCLUSION

The present study found that majority of the University of Venda students showed high perception regarding their susceptibility to the negative outcomes associated with unprotected sex, especially when they consume alcohol while engaging in sexual activities and when having multiple sexual partners. However, the students showed low perceived susceptibility to HIV transmission through the practice of oral sex. Education on the dangers of oral sex is recommended for the students.
